# Evaluation of Early-Age Concrete Compressive Strength with Ultrasonic Sensors

**DOI:** 10.3390/s17081817

**Published:** 2017-08-07

**Authors:** Hyejin Yoon, Young Jin Kim, Hee Seok Kim, Jun Won Kang, Hyun-Moo Koh

**Affiliations:** 1 Structural Engineering Research Institute, Korea Institute of Civil Engineering and Building Technology, 283 Goyangdae-Ro, Ilsanseo-Gu, Goyang-Si, Gyeonggi-Do 10223, Korea; hiyoon@kict.re.kr (H.Y.); yjkim@kict.re.kr (Y.J.K.); lagoon@kict.re.kr (H.S.K.); 2 Department of Civil Engineering, Hongik University, 94 Wausan-ro, Mapo-gu, Seoul 04066, Korea; 3 Department of Civil and Environmental Engineering, Seoul National University, 1 Gwanak-ro, Gwanak-gu, Seoul 08826, Korea; hmkoh@snu.ac.kr

**Keywords:** ultrasonic sensors, surface wave velocity, early-age concrete, compressive strength

## Abstract

Surface wave velocity measurement of concrete using ultrasonic sensors requires testing on only one side of a member. Thus, it is applicable to concrete cast inside a form and is often used to detect flaws and evaluate the compressive strength of hardened concrete. Predicting the in situ concrete strength at a very early stage inside the form helps with determining the appropriate form removal time and reducing construction time and costs. In this paper, the feasibility of using surface wave velocities to predict the strength of in situ concrete inside the form at a very early stage was evaluated. Ultrasonic sensors were used to measure a series of surface waves for concrete inside a form in the first 24 h after placement. A continuous wavelet transform was used to compute the travel time of the propagating surface waves. The cylindrical compressive strength and penetration resistance tests were also performed during the test period. Four mixtures and five curing temperatures were used for the specimens. The surface wave velocity was confirmed to be applicable to estimating the concrete strength at a very early age in wall-like elements. An empirical formula is proposed for evaluating the early-age compressive strength of concrete considering the 95% prediction intervals.

## 1. Introduction

The setting and hardening of concrete are important processes during construction work and influence the form removal time. Recent advances in design and construction technology have expanded the construction market to super-high-rise buildings and long-span bridges, which require the placement of huge quantities of concrete within the shortest time possible. Accordingly, appropriate timing for form removal is important to reduce the construction period and costs.

Concrete specifications in Korea prescribe removing the form when the concrete compressive strength reaches 5–8 MPa. For example, the Standard Concrete Specification [1], Standard Specification for Temporary Works [2], and Expressway Construction Guide Specification [3] each specify a form removal concrete strength of 5 MPa. The Manual of Concrete Practice [4] specifies a value of 8 MPa for high-strength concrete with a design strength of 40 MPa or more. On the other hand, ACI 318-14 [5], ACI 347-14 [6], and EM 1110-1-2009 [7] all state that the designer should directly decide the removal time of forms considering that “the concrete exposed by form removal shall have sufficient strength not to be damaged by deflection or twisting during removal operation.”

**Figure 1:**
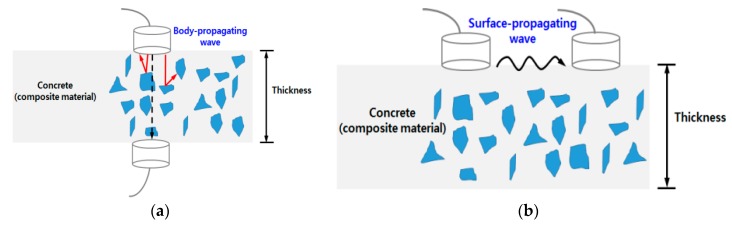
Schematic description of wave propagation in concrete: (**a**) body-propagating wave and (**b**) surface-propagating wave.

To monitor the early-age strength of concrete, various nondestructive evaluation methods such as active sensing methods using embedded piezoelectric transducers [8,9,10] and electro-mechanical impedance methods [11,12,13] have been investigated. Ultrasonic wave velocity methods are widely used to monitor the solidification of concrete because the material strength influences the propagation velocity of such waves [14]. The P-wave velocity measurement is most commonly used to evaluate the in situ concrete strength in a structure. This technique is prescribed in ASTM C597-16 [15], provided that opposite sides of the structures are accessible. However, Figure 1a shows the practical limitations of measuring the P-waves in concrete elements. The disturbance to the passing body waves by the aggregate and reinforcing elements in the concrete structure make it difficult to access opposite surfaces of some concrete structures, such as pylons and towers. Figure 1b shows the propagation of a surface wave in concrete. Surface wave-based methods have been investigated because they only need access to one side [16,17]. Research on the relationship between the surface wave velocity and concrete strength has focused on hardened concrete [18,19,20,21]. This study investigates the feasibility of using surface wave velocities to predict the strength of in situ concrete inside the form at a very early stage and the correlation between the surface wave velocity and concrete strength.

## 2. Finite Element (FE) Simulation of Surface Wave Propagation

### 2.1. Objective

**Figure 2:**
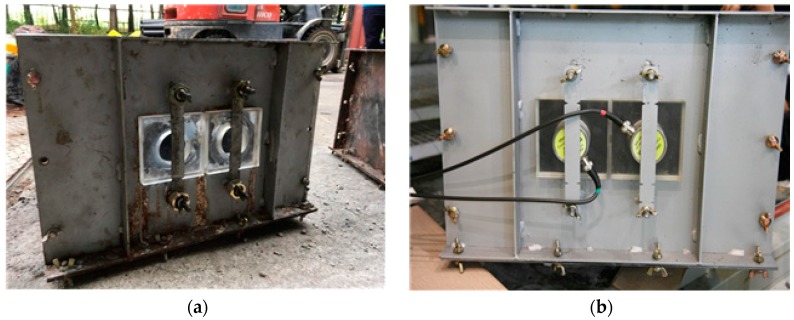
Preparation of the steel mold for surface wave velocity measurement: (**a**) mounting the acrylic panel within the steel mold before the installation of the ultrasonic transducer and (**b**) the steel mold after the installation of the ultrasonic transducer.

Stress waves are generated when pressure is induced by ultrasonic sensors attached to a concrete surface. Rayleigh waves have been adopted by many researchers for nondestructive testing purposes [18,19,20,21]. When generated in concrete, Rayleigh waves propagate along the free surface of the concrete. In this study, surface waves that propagate along the interface between concrete and formwork are needed to determine the in situ concrete strength at an early curing stage. Ultrasonic sensors were installed on the side of the concrete wall with an acrylic mounting panel in the formwork to generate and record the surface waves. Figure 2 shows the wall-side installation of the ultrasonic module. The mounting panel was used to fix the position of the sensors in contact with the concrete from the plastic state. Surface waves were generated from one of the ultrasonic sensors and traveled along the interface between the concrete and mounting panel. The waves are, in fact, Stoneley waves guided along the interface between two solids, which differ from Rayleigh waves traveling near a solid surface. Accordingly, the surface wave velocity with the mounting panel should be compared with the Rayleigh wave velocity. This was done with a FE simulation.

### 2.2. Finite Element Modeling

**Figure 3:**
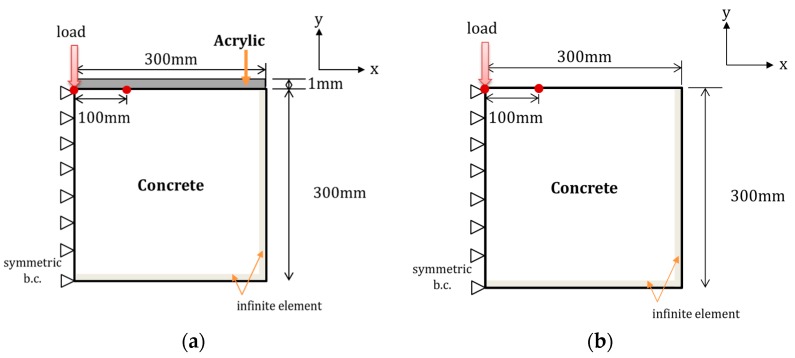
Configuration of finite element (FE) modeling: (**a**) with acrylic and (**b**) without acrylic.

**Figure 4:**
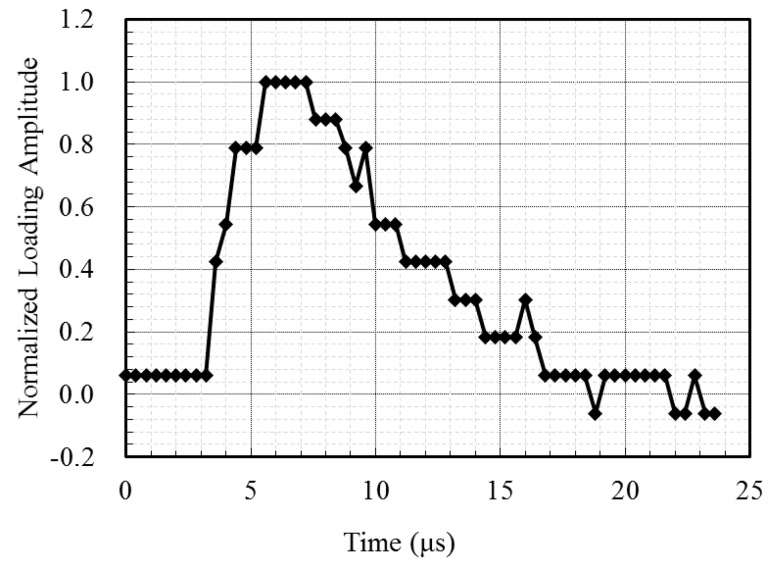
Excitation signal measured in the experimental study.

To compare the surface wave velocity with the Rayleigh wave velocity, two types of FE models were developed depending on whether acrylic was installed. Figure 3a shows the configuration of the FE model with an acrylic mounting panel, which reflects the experimental condition of this study. In this case, the surface wave leaving the transducer was transferred through the interface between the concrete and acrylic panel. Figure 3b shows the model without acrylic, where Rayleigh waves leaving the transducer were transferred along the concrete with a free surface. ABAQUS/Explicit was adopted for the FE simulation. CAX4R is a four-node, axisymmetric solid element with reduced integration and was used to model the concrete and acrylic panel. CINAX4 is a four-node, axisymmetric infinite element and was used for modeling the far edge of the concrete zone. The element size was set to 1.25 mm and the integration time step was set to 17 µs, based on the suggestion by Moser et al. [22]. During the measurement, two ultrasonic transducers were placed 100 mm apart on the same surface side of the specimen. Sensors were modeled as single nodes. The vertical displacement at the location of the other sensor, spaced 100 mm apart from loading position, was investigated. The nominal frequency of the ultrasonic waves in FE simulation is 50 kHz. The time history and nominal frequency of the waves are the same as in surface wave measurement tests. Figure 4 shows the loading signal normalized by its peak value for the simulation. The vertical displacement at the location 100 mm apart from the loading point was investigated. The signal was processed with the continuous wavelet transform to compute the travel time of the propagating surface waves.

### 2.3. Signal Processing

The surface wave velocity is determined by using the arrival time of surface waves between two ultrasonic sensors. There are several methods for determining the arrival time, such as using the first peak in the time domain [19], using the cross-correlation function [23], and using the continuous wavelet transform (CWT) [18]. The CWT allows a time-frequency analysis of a signal [24]. In contrast to the well-known Fourier transform, the wavelet-transformed signal is shown in both the frequency and time domains. The wavelet transformation is presented in Equation (1). It is a very efficient tool for calculating the arrival time of Rayleigh waves because the energy waves from the incident loading are predominantly converted into Rayleigh waves (67%) rather than P-waves (7%) or S-waves (26%) [25]. The CWT has been reported to be effective for the signal processing of Rayleigh waves [26,27]. There are numerous wavelet functions, often called the mother wavelet. In this study, a Morlet wavelet is adopted, which is a locally, periodic sinusoidal wave windowed by a Gaussian envelope:

(1) $$w\left(b,a\right)=\frac{1}{\sqrt{a}}\int_{-\infty }^{+\infty }g\left(t\right)\psi \left(\frac{t-b}{a}\right)dt$$

(2) $$\psi \left(t\right)=\exp\left(-\frac{t^{2}}{2}\right)\cos\left(5t\right)$$

**Figure 5:**
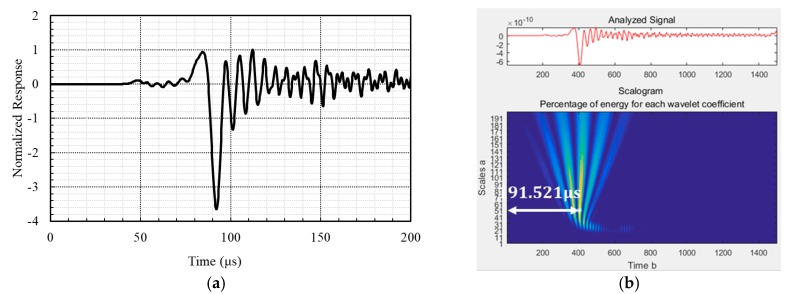
Measured time history of surface wave motion and the contour plot of the Morlet wavelet for the propagating waves. (**a**) Measured signal; (**b**) Travel time calculation with the Morlet wavelet.

Here, g(t) is the loading signal, ψ(t) is the wavelet function, *a* is a scale parameter, and *b* is a shift parameter. Equation (2) is the Morlet wavelet function. It is widely used in the time-frequency analysis of elastic waves and shows good performance in time localization [28,29]. Figure 5 shows the measured time history of surface wave motion and the contour plot of the Morlet wavelet for the propagating waves.

### 2.4. Surface Wave Velocity between the Concrete and Acrylic Layer

Table 1 presents wave velocities calculated from FE simulations. Case 1 shows the estimated velocities for the surface and Rayleigh waves. The elastic modulus and density of concrete are assumed to be 6.23 GPa and 2400 kg/m^3^, respectively. Poisson’s ratio of concrete is assumed to be 0.3 to reflect the characteristics of early-age concrete. Rayleigh wave velocity along the free surface is estimated to be 857.29 m/s, whereas the surface wave velocity between the concrete and acrylic layer is 883.96 m/s. The difference is less than 3.2%. This indicates that the acrylic panel, used as a mounting device for the ultrasonic transducer, has a negligible effect on the velocity.

Case 2 shows the surface wave velocities when considering differences in concrete density due to material uncertainty. The concrete density ranges from 2200 to 2400 kg/m^3^. The elastic modulus and Poisson’s ratio are assumed to be 6.23 GPa and 0.3, respectively. The velocity with a density of 2200 kg/m^3^ is 894.52 m/s. The higher the density is, the slower the waves propagate, as shown in Table 1. For a concrete density change of 2200–2400 kg/m^3^, the change in velocity is less than 5%.

Case 3 shows the surface wave velocities considering differences in Poisson’s ratio due to material uncertainty. Poisson’s ratio ranges from 0.16 to 0.35. The elastic modulus and density of concrete are assumed to be 6.23 GPa and 2400 kg/m^3^, respectively. The surface wave velocity with a Poisson’s ratio of 0.35 is 847.93 m/s. The lower the Poisson’s ratio is, the faster the waves propagate. For a Poisson’s ratio change of 0.16–0.35, the change in velocity is less than 5%.

## 3. Measurement of Surface Waves

### 3.1. Objective

The objective of the surface wave measurement is to evaluate the feasibility of using the surface wave velocity to predict the strength of concrete inside a form at a very early stage. A series of surface wave measurements on in situ concrete were conducted within the first 24 h after pouring by using the ultrasonic sensors. The cylindrical compressive strength test and the penetration resistance test were also performed as the surface wave velocity was measured.

### 3.2. Experimental Setup and Materials

**Figure 6:**
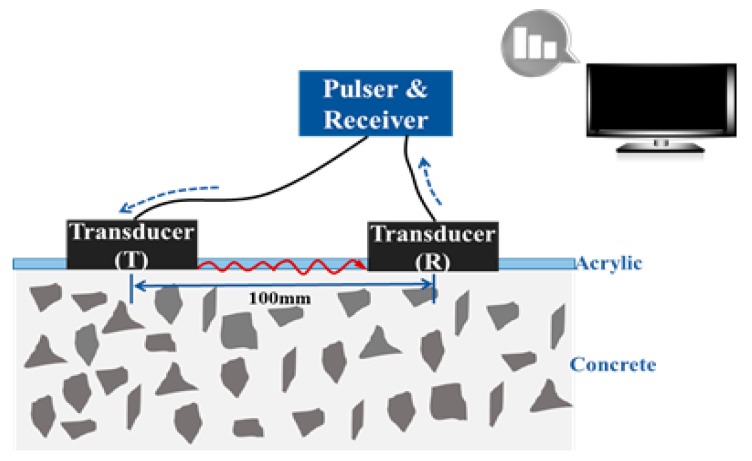
Schematic of the surface wave measurement setup.

The time-of-flight of the propagating surface waves between the concrete and mounting acrylic panel was used to derive the surface wave velocity for predicting the compressive strength of concrete inside the form. Figure 6 shows the schematic setup for the surface wave measurement: a pulser and receiver (Ultracon-3030, MKC Korea, Seoul, Korea) for generating a 600 or 1200 V signal and for measuring the received signal in the range of 1–10 MHz, a piezoelectric ultrasonic transducer pair (CT-1010, MKC Korea, Seoul, Korea) with a nominal frequency of 50 kHz, and a wireless data acquisition unit (MKDQ-710, MKC Korea, Seoul, Korea) to transfer the measured signal to the PC wirelessly. The ultrasonic sensors were installed on the same wall side of the specimen 100 mm apart. The pulser sent a short, high-voltage signal to a transmitter to cause it to vibrate at its resonant frequency. The surface wave leaving the transmitter was transferred through the surface of the specimen and arrived at the receiver. The signal was received by another transducer attached to the same surface and the velocity was calculated using the arrival time. The CWT was used to calculate the arrival time.

The mixing ratio of concrete can significantly affect the development of the concrete strength over time. Four mixture proportions were used and designated as MIX1–MIX4, given in Table 2. Because the curing temperature can also significantly affect the development of concrete strength, specimens T35, T30, T20, T15, and T05 were prepared at curing temperatures of 35, 30, 20, 15, and 5 °C, respectively. These temperatures reflect concrete construction in different seasons from summer to winter. The water to cement ratio (W/C) ranged from 35.4% to 39.2%. The sand to aggregate ratio (S/A) ranged from 42% to 46%. MIX1 and MIX2 were used in the construction of the main tower of Yi Sun-shin Bridge in Korea. MIX1 uses ordinary Portland cement (OPC) and MIX2 uses blast furnace slag cement (SC). MIX3 contains fly ash (FA). It was used in the construction of the main tower of Seohae Bridge in Korea. MIX1–MIX3 have a design compressive strength of 40 MPa. MIX4 has a design strength of 80 MPa to reflect the recent developments of high performance concrete (HPC). Table 2 presents the details of the mix proportions.

### 3.3. Experimental Procedure

**Figure 7:**
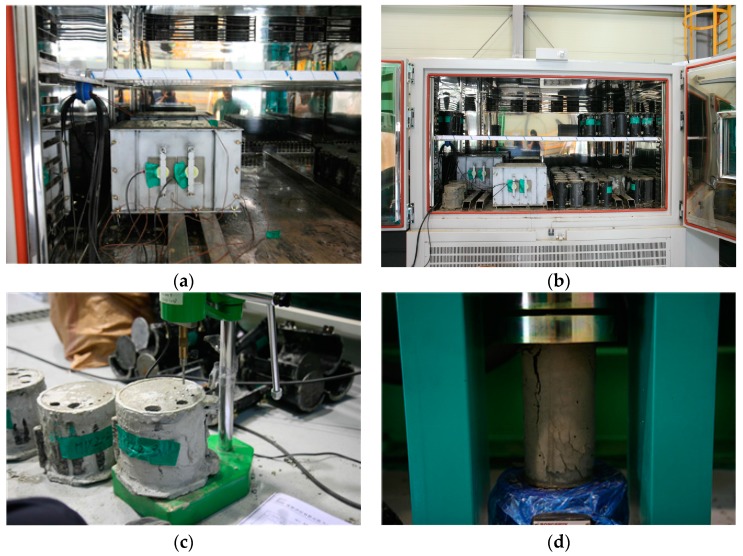
Experiments for in situ concrete at an early age: (**a**) specimen for the surface wave velocity measurement, (**b**) specimen in an isothermal control machine, (**c**) specimen for the penetration resistance test, and (**d**) specimen for the cylindrical compressive strength test.

Twenty concrete specimens were prepared to cover the various mixture proportions and curing temperatures for the surface wave velocity measurement. Freshly mixed concrete was placed in a steel mold to simulate formwork with dimensions of 400 mm × 300 mm × 300 mm as shown in Figure 7a. The surface wave measurements were conducted on the side of a wall. Two ultrasonic sensors were placed 100 mm apart. To fix the sensor positions, an acrylic plate with a thickness of 1 mm was mounted on the steel mold. The two ultrasonic transducers were then inserted into an acrylic module. After placement, the specimens were kept under isothermal conditions as shown in Figure 7b for 24 h, during which time the experiments were conducted.

Along with the surface wave velocity measurement, the penetration resistance and cylindrical compressive strength tests were conducted in accordance with KS F 2436 [30] and KS F 2403 [31], respectively. Figure 7c,d shows the two tests, respectively. The penetration resistance test was performed to determine the setting time of concrete and to investigate the validity of using the surface wave velocity for early-age concrete. The cylindrical compressive strength test was conducted to estimate strength of in situ concrete. These two tests were performed at intervals of 30 min to 1 h while the surface wave velocity was measured. Each test was repeated three times and the results were averaged.

## 4. Experimental Results

### 4.1. Surface Wave Velocity for Monitoring Early-Age Concrete

**Figure 8:**
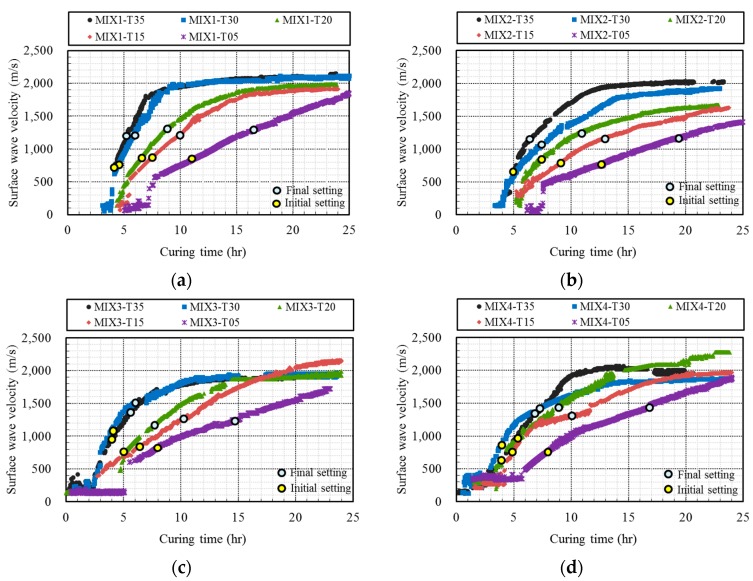
Surface wave velocities with the initial and final setting times: (**a**) MIX1, (**b**) MIX2, (**c**) MIX3, and (**d**) MIX4.

Figure 8 shows the development of the surface wave velocity with the curing age. To investigate the feasibility of using the surface wave velocity to monitor early-age concrete, the initial and final setting times were superposed. According to KS F 2436 [30], the initial and final settings of concrete are determined when the penetration resistance of a sieved mortar sample reaches 3.5 and 28 MPa, respectively. These penetration resistance values correspond to two particular practical points, which are loosely defined as the limit of handling and the beginning of mechanical strength development, respectively. The initial and final setting times determined by the penetration resistance test for the 20 concrete specimens of this study are presented in Table 3.

Observations showed that at a very early age there was considerable scatter and no well-defined trend in the computed velocities under 500 m/s. However, the plots in Figure 8 show an initial sharp increase in the velocity with age in the first few hours followed by asymptotic leveling after the casting. At the time of initial setting, the velocity development curves were already well-defined for all concrete mixtures. After the time of final setting, the velocity development curves showed asymptotic leveling. Thus, the surface wave velocity appears to be suitable for monitoring the strength gain in young concrete. A consistent surface wave velocity can be obtained from concrete after a few hours of casting and such measurements are sensitive to the developing strength up until the final setting time. Thus, the surface wave velocity is indeed suitable for monitoring the hardening process of very young concrete.

### 4.2. Correlation between the Compressive Strength and Surface Wave Velocity

**Figure 9:**
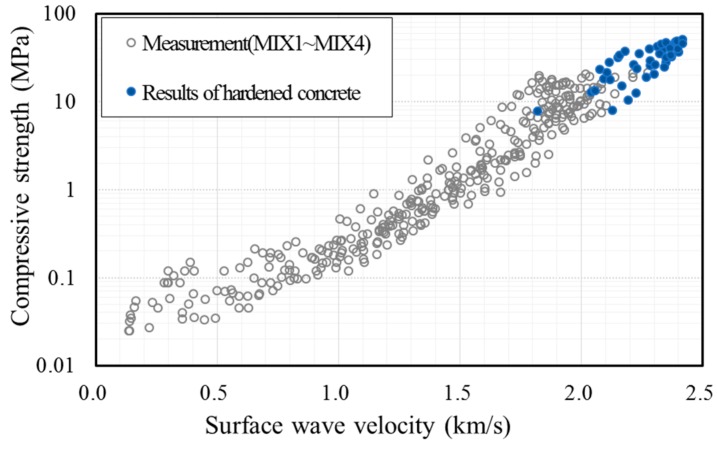
Relationship between the compressive strength of concrete and surface wave velocity; the measurement data of fresh concrete and the existing data of hardened concrete.

Figure 9 shows the measurement results for all specimens. It shows the development of the concrete strength during the first 24 h and the corresponding surface wave velocities. The surface wave velocity increased with concrete strength as the specimen hardened with age. During the 24 h, the strength of the concrete developed up to about 10 MPa and the surface wave velocity increased to around 2000 m/s.

**Figure 10:**
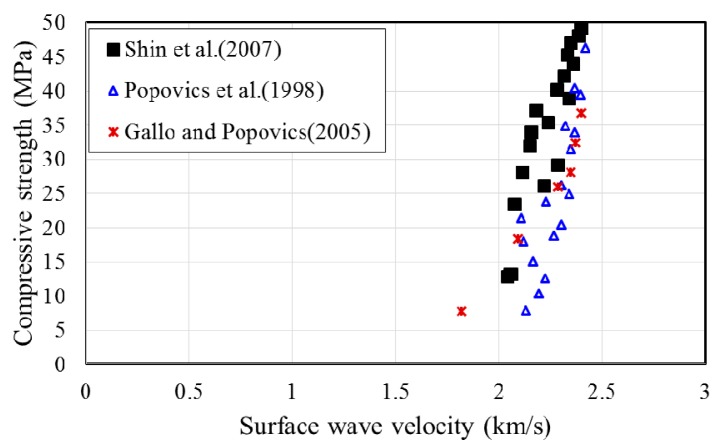
Existing data for hardened concrete.

To further examine the relationship between the surface wave velocity and concrete strength at an early age, the measurement data for hardened concrete from previous studies [18,19,20], as shown in Figure 10, are superposed in Figure 9. Shin et al. [18] considered concrete at an age of 2–28 days. Popovics et al. [19] adopted a curing age of 24 h–28 days. Gallo and Popovics [20] provided surface wave velocities for concrete aged for 7, 14, and 28 days. Figure 9 shows that the relationship between the compressive strength and surface wave velocity of early-age concrete within 24 h is consistent with that of hardened concrete obtained from previous studies using Rayleigh waves. This result demonstrates that the use of surface waves, propagating between concrete and a mounting acrylic panel, is feasible for the evaluation of the compressive strength of early-age concrete.

To find the relationship between the early-age concrete strength and surface wave velocity, theoretical and empirical formulas were investigated. For the theoretical approach, the modulus of elasticity *E* was set as proportional to the square of the pulse velocity $V_{R}$. According to ACI 318-14 [5] and KHBDC (Korea Highway Bridge Design Code, Limit State Design) [32], compressive strength is proportional to the square of the modulus of elasticity and third power of the modulus of elasticity, respectively. Accordingly, the compressive strength was represented as a power function with the surface wave velocity $V_{R}$ as given in Equation (3), which was adopted by early researchers [33,34]. However, this equation is not supported adequately by experimental results because it assumes that the material is homogeneous and linearly elastic, which concrete is not. An empirical formula has been developed by statistical means [18,35,36,37,38] and the frequently used form is given in Equation (4). Here, $f_{c}$ is the compressive strength (MPa), $V_{R}$ is the surface wave velocity (km/s), and the constants *a* and *b* are parameters determined by the least squares method to fit the measurement data in this study.

(3) $$f_{c}=a{\left(V_{R}\right)}^{b}$$

(4) $$f_{c}=ae^{bV_{R}}$$

**Figure 11:**
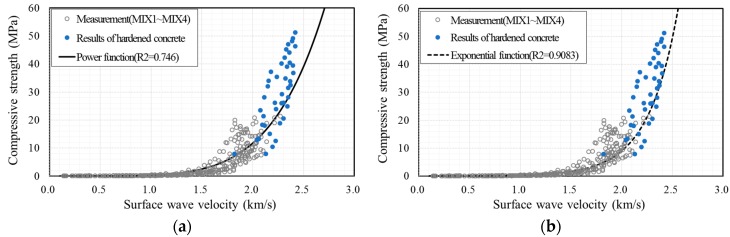
Compressive strength of concrete vs. surface wave velocity: experimental data fitted by (**a**) the power function of Equation (3) with *a* = 0.2813 and *b* = 5.3814 and (**b**) the exponential function of Equation (4) with *a* = 0.0098 and *b* = 3.412.

**Figure 12:**
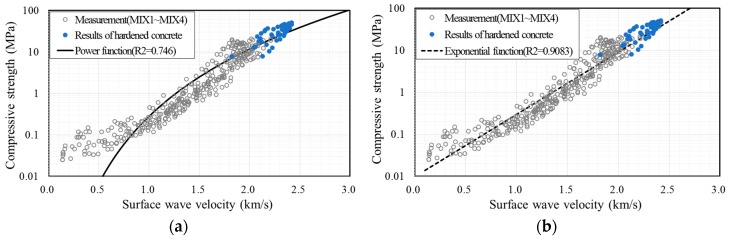
Compressive strength of concrete vs. surface wave velocity on a logarithmic scale for the vertical axis: experimental data fitted by (**a**) the power function of Equation (3) with *a* = 0.2813 and *b* = 5.3814 and (**b**) the exponential function of Equation (4) with *a* = 0.0098 and *b* = 3.412.

Figure 11 shows the fitted graphs superposed with measurement data. The data were fitted with Equation (3) in Figure 11a, while Figure 11b was fitted with Equation (4). Compressive strength increased with the surface wave velocity in each case. The coefficient of determination ($R^{2}$) is 0.746 in Figure 11a and 0.9083 in Figure 11b. Because the concrete strength for form removal is less than 10 MPa [1,2,3,4,5,6,7], the measured values need to be compared with the fitted graph in the low-strength region. Figure 12 shows the fitted graph with a logarithmic scale for the vertical axis. The measured data fit well with the empirical formula. Therefore, the following empirical formula is proposed for the relationship between very early compressive strength and surface wave velocity:

(5) $$f_{c}=0.0098e^{3.412V_{R}}$$

Equation (5) is determined by calculating the coefficients *a* and *b* of the exponential function presented in Equation (4) using a nonlinear regression method.

### 4.3. Proposed Empirical Formula to Evaluate the Compressive Strength by Using the Surface Wave Velocity

**Figure 13:**
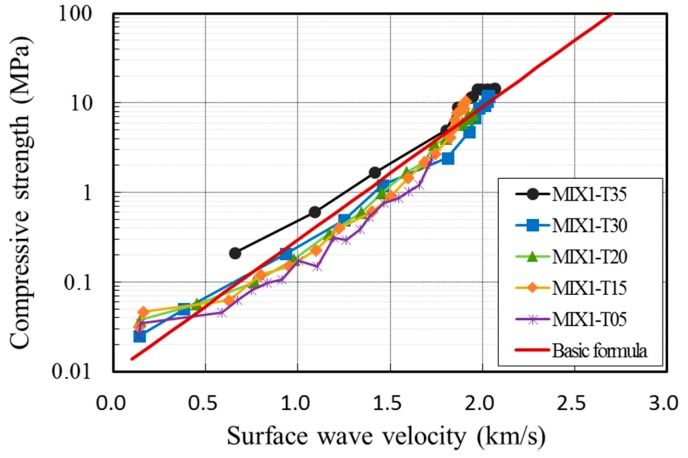
Relationship between the proposed formula and measurement data at different curing temperatures (MIX1).

Figure 13 shows the data of compressive strength and surface wave velocity from MIX1 with the proposed formula of Equation (5). In general, the recorded compressive strength was higher than the value calculated by the formula at 30 and 35 °C and lower at 5 °C. This indicates that the curing temperature has an effect on the relationship between the early-age compressive strength and surface wave velocity. To consider the influence of the curing temperature on the proposed formula, the equation was modified to include the coefficient of temperature, k, as in Equation (6). The measurement data for each mixture were divided into three subgroups. Group A consisted of data measured at temperatures between 30 and 35 °C, to reflect summer construction. Group B consisted of data measured at temperatures between 15 and 20 °C and was referred to as room-cured specimens. Group C consisted of data at 5 °C to reflect winter construction.

(6) $$f_{c}=0.0098ke^{3.412V_{R}}$$

**Figure 14:**
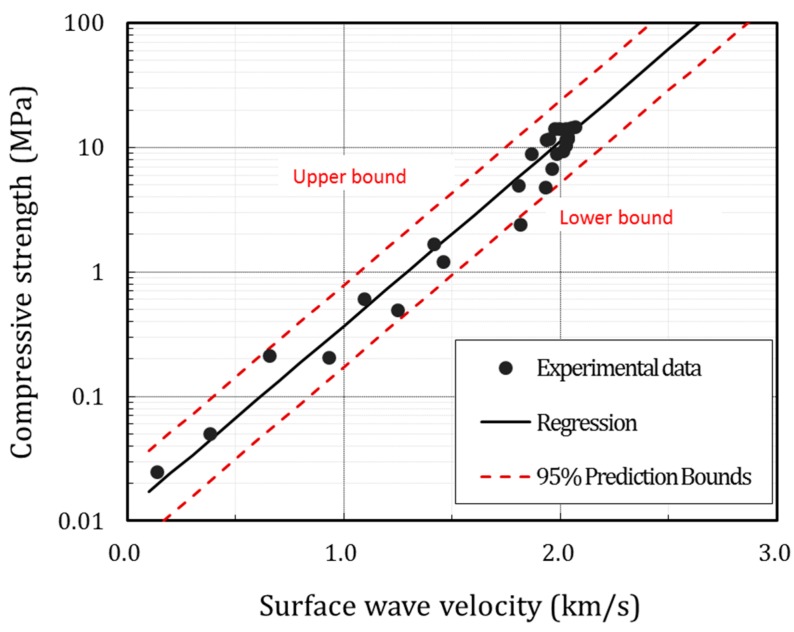
Fitted formula with the 95% prediction bound (MIX1).

Figure 14 shows the line of best fit with a 95% prediction interval for group A with MIX1. Considering the field applicability, the coefficient of temperature in Equation (6) was determined with the lower bound limit. The coefficient of temperature for temperature groups A, B, and C for four mixture proportions in this study are listed in Table 4.

## 5. Conclusions

In this study, the surface wave velocity measured with ultrasonic sensors was verified to be applicable to the monitoring of the solidification of concrete inside the form and an empirical formula was developed for evaluating early-age concrete strength according to the surface wave velocity. A pair of ultrasonic sensors was installed on the same side prior to placement. The sensors were used to measure the surface wave velocity of concrete. An acrylic panel was used to fix the position of the sensors and to increase the applicability on structures such as walls and towers. An FE simulation was conducted to compare the surface wave velocity with the Rayleigh wave velocity and the difference between them was less than 5%, even when material uncertainties were considered. The surface wave velocity for early-age concrete inside a form were comparable with the measured Rayleigh wave velocity for hardened concrete from previous studies. A series of experiments including surface wave velocity measurement, a cylindrical compressive strength test, and a penetration resistance test were conducted on in situ concrete during the first 24 h after placement. The penetration resistance test showed that a consistent surface wave velocity can be obtained prior to the initial setting and the measurement was sensitive to the developing strength up until the final setting time. Thus, the surface wave velocity is suitable for monitoring the hardening process of very young concrete. Based on the relationship between the measured surface wave velocity and the corresponding cylindrical compressive strength, an empirical exponential function was developed. The formula was obtained using all measurement data in this study. Then, a temperature coefficient, to account for the effect of construction in different seasons, was added to the formula considering the 95% prediction bound.

The proposed formula can be used with the measured surface wave velocity for concrete inside a form to predict the compressive strength of concrete. The method can help with determining the appropriate form removal time for curing concrete, thereby contributing to the reduction of a construction period. The method has been developed based on the mix proportion of concrete used in this study and, therefore, further research is needed to investigate the applicability of the method for various mix proportions of concrete.

## Figures and Tables

**Table 1:** Comparison of wave velocity from FE simulation.

Case	Specification		Start Time (µs)	Arrival Time (µs)	Velocity (m/s)	Difference (%)
Case 1: effect of acrylic layer	Rayleigh wave (without acrylic)		3.2	119.846	857.29	100
	Surface wave (with acrylic)		3.2	116.327	883.96	103.1
Case 2: effect of concrete density	Density (kg/m^3^)	2200	3.2	114.992	894.52	100
		2250	3.2	116.292	884.24	98.9
		2300	3.2	117.577	874.30	97.7
		2350	3.2	118.550	866.93	96.9
		2400	3.2	119.805	857.60	95.9
Case 3: effect of Poisson’s ratio	Poisson’s ratio	0.16	3.2	116.327	883.96	100
		0.20	3.2	117.364	879.05	99.4
		0.28	3.2	119.454	875.93	99.1
		0.30	3.2	119.805	872.31	98.7
		0.35	3.2	121.134	860.55	97.4

**Table 2:** Mixture proportions per cubic meter for the surface wave velocity measurement, cylindrical compressive strength test, and penetration resistance test.

Index	Type		W/C (%)	S/A (%)	Unit Weight (kg/m^3^)					Curing Temperature (°C)	Design Strength (MPa)
					Fly Ash	Super-Plasticizer	Cement	Sand	Gravel		
1	OPC	MIX1-T35	35.4	46	-	4.75	475	760	935	35	40
2		MIX1-T30								30	
3		MIX1-T20								20	
4		MIX1-T15								15	
5		MIX1-T05								5	
6	SC	MIX2-T35	35.4	46	-	4.75	475	760	935	35	40
7		MIX2-T30								30	
8		MIX2-T20								20	
9		MIX2-T15								15	
10		MIX2-T05								5	
11	FA	MIX3-T35	38	42	104	7	419	672	932	35	40
12		MIX3-T30								30	
13		MIX3-T20								20	
14		MIX3-T15								15	
15		MIX3-T05								5	
16	HPC	MIX4-T35	39.2	45	-	4.2	420	674.3	827.9	35	80
17		MIX4-T30								30	
18		MIX4-T20								20	
19		MIX4-T15								15	
20		MIX4-T05								5	

**Table 3:** Initial and final setting times determined by the penetration resistance test for 20 concrete specimens.

Type	Required Time (h)		Type	Required Time (h)	
	Initial Setting	Final Setting		Initial Setting	Final Setting
MIX1-T35	3.7	5.3	MIX3-T35	3.9	5.8
MIX1-T30	4.6	6.1	MIX3-T30	4.2	6.3
MIX1-T20	6.4	8.8	MIX3-T20	5.0	7.8
MIX1-T15	7.3	10.2	MIX3-T15	6.6	10.2
MIX1-T05	11.2	16.6	MIX3-T05	8.1	14.7
MIX2-T35	5.0	6.5	MIX4-T35	3.6	6.9
MIX2-T30	4.9	7.3	MIX4-T30	4.0	7.2
MIX2-T20	7.4	10.9	MIX4-T20	5.4	9.0
MIX2-T15	9.2	13.0	MIX4-T15	4.8	10.1
MIX2-T05	12.7	19.4	MIX4-T05	8.1	16.7

**Table 4:** Temperature coefficient, k, for mixtures 1–4.

Type	Temperature (°C)	*K*	*R*^2^	Type	Temperature (°C)	*K*	*R*^2^
MIX1	30–35	1.2394	0.874	MIX3	30–35	2.4437	0.925
	15–20	1.0955	0.901		15–20	1.1207	0.567
	5	0.52677	0.934		5	0.64678	0.982
MIX2	30–35	1.1981	0.939	MIX4	30–35	2.3002	0.846
	15–20	0.83825	0.95		15–20	1.0433	0.943
	5	0.40681	0.961		5	0.41227	0.975
